# Albert J. Evans

**Published:** 1913-04

**Authors:** 


Albert J. Evans, Hahnemann of Philadelphia, 1871, died at Pasadena February 28, aged 68 (88 in other notices). He was credited to Lockport, N. Y., in Polk, but left that town for Fredonia about twenty years ago and left Fredonia for California, on account of failing health, several years ago.

